# A Case Report on Bovine Colostrum as a Potential Therapeutic Agent Alternative to Treat Gastrointestinal Complications of Common Variable Immunodeficiency

**DOI:** 10.7759/cureus.25594

**Published:** 2022-06-02

**Authors:** Ganesh M

**Affiliations:** 1 Gastroenterology, SG Gastro Care, Coimbatore, IND

**Keywords:** bovine colostrum, intravenous immunoglobulin therapy, gastrointestinal infection, primary immunodeficiency, hypogammaglobulinemia, common variable immunodeficiency

## Abstract

Common variable immunodeficiency (CVID) or acquired hypogammaglobulinemia is one of the common forms of primary immunodeficiency, which primarily affects the respiratory tract, but is often associated with gastrointestinal complications. The pathophysiology is not fully understood, making a diagnosis of CVID difficult. The low levels of IgG and IgA and defective B cells make intravenous immunoglobulin (IVIG) therapy the mainstay of management. The present article describes the journey of a 41-year-old male suffering from CVID and severe recurrent uncontrollable gastrointestinal infections requiring Intravenous immunoglobulin therapy, which was successfully substituted with an alternative oral Hyperimmune Bovine Colostrum and Zinc combination for long term gastrointestinal disease control in a more affordable manner.

Keywords: Common variable immunodeficiency, Hypogammaglobulinemia, Primary Immunodeficiency, Gastrointestinal infection, Intravenous Immunoglobulin Therapy, Bovine Colostrum.

## Introduction

Common variable immunodeficiency is an immunodeficiency disease affecting young adults and children and is largely underdiagnosed. It is sporadic but may also be inherited [1]. Common variable immunodeficiency (CVID) is characterized by a decrease in immunoglobulin levels, predominantly IgG and IgA levels, and is associated with recurrent respiratory, gastrointestinal, autoimmune, inflammatory bowel disease granulomatous diseases [2]. CVID is also associated with genetic mutations, disturbed B cell homeostasis with reduced or absent memory B cells, increased CD21(low) B cells, transitional B cell populations, and T cell functional defects. Diagnosis requires reduced levels of at least two immunoglobulin isotypes: IgG with IgA and/or IgM and impaired specific antibody response to vaccines [1]. Hence immunotherapy is the mainstay of treatment for CVID, but it is also expensive compared to other alternative therapies [3]. We describe a case where CVID diagnosed patient was managed efficiently and cost-effectively with a novel and potential therapeutic approach by Bovine colostrum. Bovine colostrum has shown favorable outcomes in Gastrointestinal disorders due to secondary immunodeficiencies [4].

## Case presentation

A 41-year-old married male was referred after unsuccessful treatment by a general physician for recurrent gastroenteritis, with complaints of a variable to the large volume of watery stools without blood or melena, associated with intermittent abdominal pain and cramps. Additional complaints included post-prandial abdominal pain, recurrent vomiting, loss of appetite, fatigue, myalgia, polyarthritis, and gradual weight loss of 25 kg over 10 years. The patient’s complaints started 10 years back with intermittent diarrhea, which had worsened in the past year despite avoiding dairy products and food restrictions. His past medical history included pulmonary Tuberculosis at the age of five, which was managed successfully by a local private hospital in Coimbatore, and recurrent allergic rhinitis managed by his General Physician. There was no evidence of any hereditary immune deficiency or autoimmune disorder in his family history.

**Figure 1:**
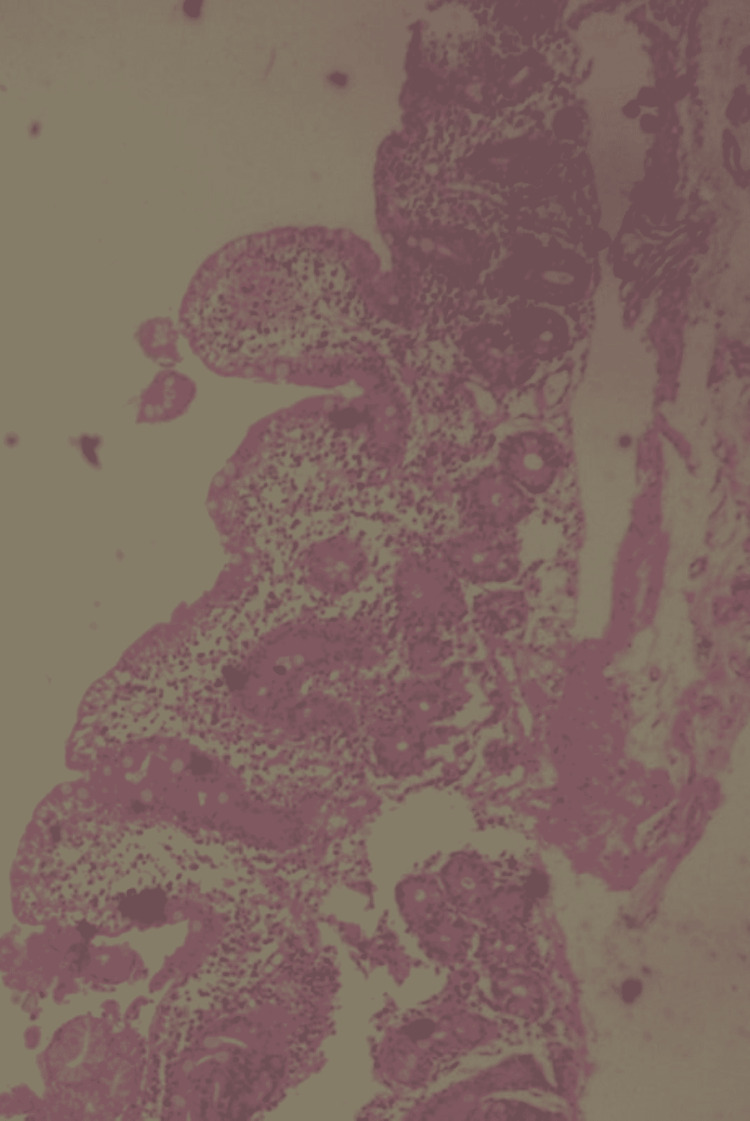
Low power microscopic view of the duodenal mucosal biopsy showing villous atrophy

**Figure 2:**
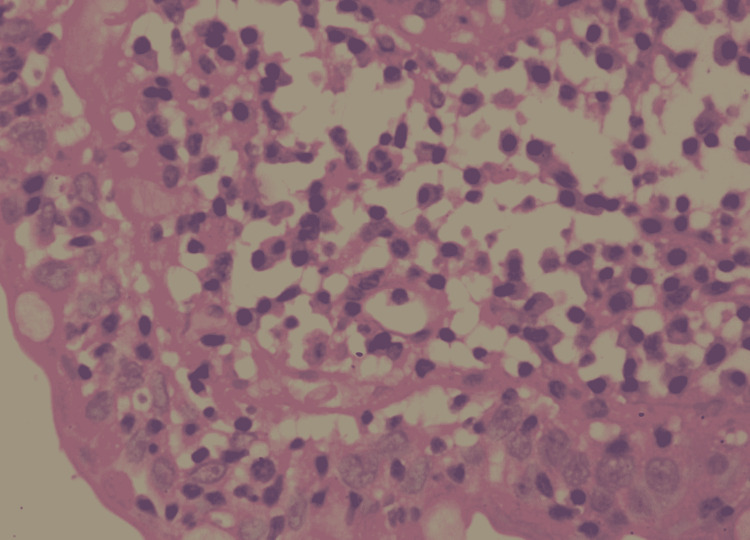
High power microscopic view of the duodenal mucosal biopsy showing villous atrophy

On admission, the patient was emaciated, weighing only 29 kg; general physical examination revealed tachycardia and tachypnoea with diffuse abdominal tenderness and distention. There was pedal edema and sluggish reflexes. Simultaneously, routine laboratory investigation revealed low Hb and high inflammatory markers ESR 106 mm/hr and CRP 36.8 mg/dl. WBC 12,600 cells/cumm, sodium 130 mmol/ml, potassium 2.9 mmol/ml. Chest x-ray showed bronchopneumonia. The stool routine showed entamoeba histolytica cysts. Additional special tests revealed low Vit D, B12, and Folic Acid levels and negative Elisa for HIV and hypogammaglobulinemia with IgG and IgA. A negative Mantoux test ruled out Tuberculosis. USG abdomen revealed mild ascites and hepatosplenomegaly; Endoscopy (OGD and Colonoscopy) revealed oesophageal candidiasis and multiple antral ulcers and erosions. The biopsy revealed (1) nonspecific colitis, (2) focal duodenal villous atrophy, and (3) h-pylori-positive gastritis. (Figure 1, 2) Anti TTG antibodies were negative.

The patient on admission was rehydrated, and electrolyte imbalance was corrected. He received intravenous ceftriaxone and metronidazole. In the subsequent days, he was closely monitored, and the antibiotic was changed to meropenem, as pyrexia was not controlled. Over a week, his condition gradually improved. The special serum immunoglobulins investigation results showed low IgG levels of 0.2 g/L (6-16) and IgA 0.1g/L (0.8-2.8), while IgM, D, and E were within the normal range. Based on the chronic history and low levels of IgG and IgA, a diagnosis of CVID was made.

The patient was discharged with every 3-weekly Intravenous immunoglobulin (IVIg) as a supplement to low-level IgG and IgA; he gained 20 Kg of weight during the follow-up period. After three months of follow-up, the patient stopped taking IVIg immunoglobulins due to the cost burden, which led to a relapse of GI symptoms, respiratory symptoms, and significant weight loss. The patient was prescribed oral antibiotics for two weeks with nutritional supplements. Hyper Immune Bovine Colostrum with Zinc (Enactis Healthcare Private Ltd.) was added to replace immunoglobulin therapy for gastrointestinal symptoms.

He started showing clinical improvement after two months of follow-up. At 20 months of follow-up, clinically, his diarrhea ultimately settled with a marked reduction in respiratory symptoms along with a 20kg weight gain. His biochemical parameters improved to near normal values except for the immunoglobulin levels, which remained the same as the base level.

## Discussion

Common variable immunodeficiency disorder (CVID) is defined [2] by markedly reduced serum concentrations of immunoglobulin IgG, in combination with low levels of IgA and/or IgM, Poor or absent antibody response to infection or immunization, or both, and An absence of any other defined immunodeficiency state.

Variable immunodeficiency refers to the heterogeneous clinical manifestations of this disorder, which include recurrent infections, chronic lung disease, autoimmune disorders, gastrointestinal disease, granulomatous disease, and heightened susceptibility to lymphoma. The hallmark immune defect in CVID is defective B cell differentiation into plasma cells, with the impaired secretion of immunoglobulin. B cell maturation is defective in patients with CVID, and the toll-like receptor (TLR) function on both B cells and dendritic cells is impaired [5-7].

CVID affects as many as 1 in 25,000 individuals [5]. There is some evidence of higher prevalence among individuals of northern European descent. Following gastrointestinal manifestations can be seen in descending order of frequency Inflammatory bowel-like disease, Sprue-like illness with flat villi, Nodular lymphoid hyperplasia, Pernicious anemia, Bacterial overgrowth, Protein-losing enteropathy, Nonspecific malabsorption, and Gastrointestinal lymphoma. Patients with deficient total memory B cells and low switched memory B cells appear more likely to develop malabsorption or chronic diarrhea than patients with normal memory B cells [6].

Chronic giardiasis causing refractory diarrhea, malabsorption, or weight loss has been reported in patients with CVID [7-9]. Other infections causing chronic diarrhea include cytomegalovirus, or rarely, cryptosporidium or norovirus [7].

Approximately one-third have chronic lung disease by the time of diagnosis, either bronchiectasis or restrictive or obstructive lung disease, which can lead to recurrent hospitalization and morbidity, other causes of hypogammaglobulinemia has to be excluded, including drug-induced - glucocorticoids, immunosuppressants, hematological malignancies - CLL, lymphoma, multiple myeloma, Waldenstrom’s macroglobulinemia, thymoma. The cornerstone of therapy is immune globulin replacement, which has dramatically altered the clinical course of CVID by reducing the burden of recurrent infections and subsequent complications. However, this has to be administered at 500mg/kg every three to 4 weeks [10,11].

In the present case report, a young male with chronic diarrhea, recurrent respiratory tract infections, and weight loss without any significant family history. Based on all the routine and specific tests and the positive hypogammaglobulinemia with IgG and IgA, a diagnosis of common variable immunodeficiency syndrome was confirmed. He was initially treated with Iv immunoglobulins for a few months, with improvement in his clinical status. However, due to his economic condition, he could not continue the therapy, and two months after stopping the therapy, severe gastrointestinal symptoms started recurring. Long-term IVIg is not an economical option for many patients due to out-of-pocket expenses and lack of insurance [12].

In a first of its kind, a new oral drug Hyper Immune Bovine Colostrum with Zinc (Enactis Healthcare Private ltd) 1 capsule three times a day, was started replacing IVIg as a viable economic alternative for severe gastrointestinal symptoms in this CVID patient. Bovine colostrum is rich in proteins and immunoglobulins, colony-stimulating factors, transforming growth factors, anti-inflammatory cytokines, interferon-gamma, and proline-rich polypeptides lactoperoxidase-thiocyanate xanthine oxidase and peroxidase enzymes, vitamins minerals, amino acids, essential oils, lactoferrin, lysozyme, trypsin, and orotic acid. These components reduce the inflammatory activity and exert a trophic effect on the cells. Lactoferrin in the colostrum is converted to lactoferricin B, killing gram-negative bacteria. Various studies have proven these findings [13]. All these mechanisms in colostrum reduce the inflammatory activity and leaky gut and prevent the entry of the bacterial endotoxin and lipopolysaccharides into portosystemic circulation [14,15].

We presume that immunoglobulins, trophic factors for the cells, and various factors that reduce the inflammation, infection, and leaky gut in bovine colostrum capsules were responsible for the positive outcome in this case. The sustained efficacy and control of disease symptoms, the convenience of oral therapy, and no reported adverse event over two years of therapy in this patient make it a viable alternative to IVIG for severe gastrointestinal manifestations of CVID. Further data and clinical trials are required to generate more evidence.

## Conclusions

The case presented here emphasizes the need for increased awareness regarding CVID in patients with an endless combination of respiratory and gastrointestinal diseases. Early strong suspicion plays a vital role in diagnosing and preventing significant morbidity and mortality due to CVID. IVIG provides a clinical improvement in these patients; our case study, for the first time, has shown that a more pharmacoeconomic alternate such as BC-Z (Enactis -Hyper Immune bovine colostrum with zinc supplement) can be substituted for IVIG in gastrointestinal predominant infections and diseases in CVID patients, to boost the local gut immunity and to improve gastrointestinal symptoms. This approach can reduce IVIG use as a cost-saving economical measure in such cases.
